# Harnessing *Salmonella* as a potent vaccine delivery platform: Targeted HA/NA epitope presentation via alphavirus RdRp-driven expression to boost immune efficacy

**DOI:** 10.1016/j.mtbio.2025.102224

**Published:** 2025-08-21

**Authors:** Jun Kwon, Amal Senevirathne, John Hwa Lee

**Affiliations:** Laboratory of Veterinary Public Health, College of Veterinary Medicine, Jeonbuk National University, 79 Gobong-ro, Iksan, 54596, Jeollabuk-do, Republic of Korea

**Keywords:** *Salmonella* delivery system, Epitope engineering, Hemagglutinin, Neuraminidase, RNA polymerase-driven expression

## Abstract

In this study, we developed an enhanced *Salmonella*-based delivery platform for safe and efficient intracellular plasmid delivery, incorporating rationally designed influenza antigen epitopes to boost protective immunity. Two key viral antigens, hemagglutinin (HA) and neuraminidase (NA), were selected for their complementary roles in the viral life cycle and cloned into a eukaryotic expression system driven by RNA-dependent RNA polymerase to ensure robust intracellular expression. HA was engineered to retain conserved epitopes within the stalk region and receptor binding site (RBS), while NA was stabilized in a trimeric conformation via C-terminal fusion with the T4 phage fibritin domain. The two antigens were linked by a furin cleavage site to promote efficient intracellular processing and antigen presentation. Immunization and subsequent challenge studies in mice demonstrated strong antigen-specific humoral and cellular immune responses, with activation of both Th1 and Th2 pathways. Notably, antibody subclass profiling indicated a Th1-skewed response beyond 21 days post-infection. Assessment of long-term immunity confirmed that even 42 days after booster vaccination could successfully respond to vaccinated antigens, elucidating the effectiveness of the *Salmonella* system. Vaccinated mice were protected from lethal challenge with multiple influenza strains, maintaining body weight and exhibiting significantly reduced viral loads in lung tissues, highlighting the applicability of our epitope-based vaccine model. These findings underscore the potential of the *Salmonella*-mediated vaccine strategy described here as an effective and scalable approach for influenza immunization.

## Graphical abstract

**Figure:**
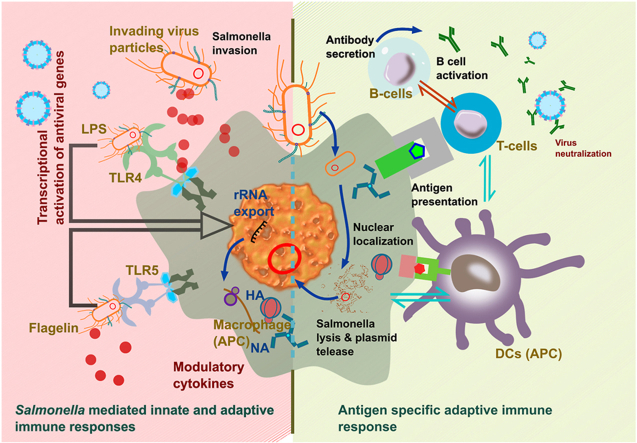

## 1 Introduction

Developing annual vaccine plans against influenza is critically important for safeguarding public health against seasonal disease spread [1,2]. Influenza is a highly contagious disease with substantial morbidity and mortality, especially among vulnerable populations such as the elderly, the young, and individuals with chronic health conditions [2,3]. Effective vaccine planning plays a key role in mitigating the impacts of seasonal influenza in many parts of the world [4,5]. Frequent re-evaluations of these vaccine antigens are essential to maintain vaccine efficacy according to field match strains [6]. Development of effective broad-spectrum vaccines against influenza is of paramount importance but remains a challenging task as protective antigens such as hemagglutinin remain highly variable among members of the influenza virus pool.

Multivalent antigens used in commercial vaccines may often protect against multiple influenza strains. However, a fundamental shift in influenza vaccination strategies is necessary for more efficient distribution of vaccines to the growing global population [7]. Dependence on embryonated eggs [8] or advanced technologies, such as mRNA-based vaccines [9] or viral vector vaccines [10], may present limitations during periods of high demand, particularly in the event of epidemics or pandemics. This highlights the need for vaccination strategies that are effective, scalable, stable, and cost-efficient, rather than those that require extensive resources. Bacteria-mediated immunotherapy is a promising approach to achieve the vaccine strategy goals outlined above. Attenuated bacterial species, such as *Salmonella*, specifically target the host's antigen-presenting cells, triggering both mucosal and systemic cell-mediated immune responses. Additionally, antigens expressed within *Salmonella* remain thermodynamically stable for extended periods, reducing the burden of strict preservation conditions for vaccines during deployment until they reach their intended cellular targets. Furthermore, the broad immune activation driven by multiple pathogen-associated molecular patterns (PAMPs) can enhance the effects of virus-specific antigens, potentially overcoming the challenges posed by viral diversity.

A major concern when using bacterial species such as *Salmonella* is safety, as some of the pathogenic strains elicit extremely potent immune responses, which can be detrimental to the host animal. As a vector system, a balance between virulence and attenuation is essential to mount an immune response that is strong enough to fight viral infection yet safe enough to avoid deterioration of the health of the animal host. Meeting these objectives requires careful curation of virulence-related genes, which are organized into several pathogenicity islands (SPIs) within the *Salmonella* genome [11]. We developed a significantly attenuated *Salmonella* strain featuring favorable properties, such as hyper-invasiveness in epithelial cells and antigen-presenting cells but with rapid clearance and without transformation into a chronic infection. We engineered these features through specific deletions of two virulence-related genes, *lon* and *cpxR* (SPI-1) [[12], [13], [14], [15]]. Absence of the global virulence regulator *lon* results in hyperinvasive *Salmonella*, yet the virulence cannot be sustained due to compromised membrane stress tolerance owing to lack of the *cpxR* gene.

Once *Salmonella* enters a cell, its natural tendency is to remain and mature in *Salmonella-*containing vacuoles (SCVs) [16], which serves as a bottleneck concerning the delivery of therapeutic plasmids into the cytoplasm. To limit *Salmonella* occupancy in SCVs, we deleted another gene, *sifA*, responsible for an SPI-2 effector protein [17]. To immortalize the therapeutic plasmids in *Salmonella,* additional deletion of aspartate semialdehyde dehydrogenase (*asd*) was conducted, allowing *Salmonella* to retain *asd-*complemented plasmids indefinitely without requiring selective pressure imposed by antibiotic markers. Based on these features, our novel *Salmonella* system is unique in design and highly applicable for influenza antigen delivery to obtain a multifaceted immune boost.

Despite broad utilization, modern anti-influenza vaccines harness only 40–60 % of protection efficacy even with good antigenic similarity between the vaccine antigens and circulating viruses [7]. This highlights certain insufficiencies of current vaccine designs against influenza infections. We suggest consideration of *Salmonella-*mediated anti-influenza vaccine models as next-generation vaccines. The interaction between *Salmonella* and the host immune system elicits a multi-lateral and holistic immunity compared to that conferred by a singular antigen system. Furthermore, the *Salmonella* system enables delivery of multiple antigens, resulting in more robust antiviral immunity that could enhance the protective efficacy of the current system. We leveraged two protective antigens, hemagglutinin (HA) and neuraminidase (NA), in a rational vaccine design to mitigate existing issues in influenza vaccinology. When comparing HA and NA sequences among viruses, the antigens demonstrate significant sequence variations. However, the rate of mutations is higher for HA than NA [18]. The receptor binding site (RBS) of HA is especially variable, yet its presence is essential for protection efficacy. To overcome this complexity, we hypothesized that application of conserved epitopes from both HA and NA in a specially designed trimer confirmation would be advantageous for the *Salmonella* system [19]. We reasoned that combined anti-viral and *Salmonella-*mediated innate and adaptive immunity would activate the immune system by suppressing influenza viral activity within the immunized host. Furthermore, elicitation of highly specialized epitope-driven immune responses could be useful to expand the breadth of protection while avoiding the body's responses toward less important regions of a protein immunogen. To further promote antigen processing and presentation within APCs, we fused HA and NA epitopes using a furin protease cleavage site, and the C-terminus of the NA segment was connected to *Escherichia coli* T4 phage fibritin, which folds into a nanoscopic trimer structure [20]. The entire construct was synthesized and cloned into an RdRp-driven eukaryotic expression system that facilitates cytoplasmic mRNA amplification [21]. By compiling two antigen systems into a single expression system, we facilitate balanced expression of both antigens by minimizing the bias towards any single antigen delivered by *Salmonella*. Since the antigens are expressed within the eukaryotic host cell, this system permits natural post-translational modifications that could be essential for a robust immune response.

In the current study, we designed and investigated a new *Salmonella*-mediated anti-influenza HA-NA epitope vaccine construct in a mouse-challenge model and assessed immunological protection against homologous H1N1 challenge. Our system was not only efficient at antigen presentation but also resulted in significant protection in immunized mice. We demonstrated that anti-sera had potential cross-protection efficacy against other related influenza H1N1 strains. Our *Salmonella*-mediated anti-influenza model is a viable option for rapid vaccine development and is capable of stimulating a multifaceted immune boost against seasonal influenza. We further propose that the efficiency of epitope vaccines can be increased by combining them with the *Salmonella* system for a synergistic effect with appealing benefits such as stronger and longer-lasting immune responses against problematic viral diseases such as influenza.

## 2 Materials and methods

### 2.1 Bacterial strains, plasmids, and primers

Bacterial strains, plasmids, and primers used in the current study are presented in Table S1. All bacterial strains were routinely cultured in Luria-Bertani medium (LB; BD, Franklin Lakes, NJ, USA) with vigorous shaking at 37 °C. Bacterial strains lacking aspartate dehydrogenase (*asd*) were cultured by supplementing diaminopimelic acid (DAP) at a 50 μg/mL concentration. Appropriate antibiotics were utilized whenever necessary.

### 2.2 Cell lines and virus strains

HeLa (CCL-2), RAW 264.7 (TIB-71), and Madin-Darby canine kidney (MDCK) cell lines (CCL-34) procured from ATCC were maintained in Dulbecco's modified Eagle's medium (Lonza, Basel, Switzerland) supplemented with 10 % fetal bovine serum (Gibco, Waltham, MA, USA), and 100 units/mL penicillin and 100 μg/mL streptomycin at 37 °C in a humidified, 5 % CO_2_ atmosphere.

For the viral strains, H1N1 strains [A/H1N1/pdm09 (NCCP43399 and NCCP43400), A/Brisbane/59/2007 (NCCP42464), A/Korea/01/09 (NCCP43021), and A/PR/8/34] provided by the National Culture Collection for Pathogens (NCCP, Cheongju, Korea) were propagated in ten-day incubated embryonated specific pathogen-free (SPF) chicken eggs. The viral strains were inoculated in the allantoic cavity of eggs while maintaining sterile conditions. Eggs were incubated at 35 °C under humidified conditions for 48 h, and the allantoic fluid was collected. The collected fluid was aliquoted and the TCID50 titers were determined by the Reed-Muench method using MDCK as the host cell line.

### 2.3 Experimental animals and ethics statement

Five-week-old, SPF female BALB/c mice (N = 25, n = 7) were purchased from Koatech (Pyeongtaek, Gyeonggi-do, South Korea). Sterilized food and water were provided *ad libitum*. A 12-h day and night cycle was maintained. Mice were housed for one week of adaptation, and at six weeks old, were utilized in the immunization procedure. Animal experiments were approved by the Jeonbuk National University Animal Ethics Committee (NON2022-024-002) under the Korean Council on Animal Care and the Korean Animal Protection Law, 2001, Article 13, and undertaken at the animal experimental facility at the College of Veterinary Medicine, Jeonbuk National University.

### 2.4 Epitope vaccine design and construction

Sequence acquisition for influenza A (H1N1) was conducted using NCBI's Influenza Virus Resource Database [22]. HA and NA sequences from the Northern temperate region, collected after the year 2000, were selected based on mutagenic variations. Nineteen HA and 82 NA full sequences were retrieved and aligned against reference sequences to identify conserved regions. Consensus sequences were developed using the COBRA method [23], ensuring that the essential domains for immune response remained intact. The HA sequence (144 AA) included the globular head, a stalk region for stability, and key epitopes (AA 159–167, 188–199, 245–251, 263–274, 319–365) with the GLF cleavage site. The NA sequence (54 AA) focused on conserved positions (AA 214–238) with proven protective epitopes. To enhance antigen presentation, a T4 fibritin trimerization domain was fused at the NA C-terminus. HA and NA sequences were linked via a furin cleavage site (RARR), reverse-translated, and synthesized (Cosmogenetec, Seoul, South Korea) for cloning.

### 2.5 Bioinformatics analysis

The linear B and T cell epitopes of the consensus sequence were predicted using the BepiPred Linear Epitope Prediction tool of the Immune Epitope Database (IEDB) [24]. The sequence was also submitted to NetMHCpan-4.1 [25] and NetMHCII-2.3 servers to predict MHC binding peptides [26]. Following the analysis of the consensus sequence, we harvested epitope regions that were highly conserved and immunogenic. The newly designed epitope sequences were confirmed for structural stability. Model accuracy was assessed by generating Ramachandran plots and validated using the PROCHECK server at the University of California, Los Angeles, CA, USA [27,28]. The antigenicity of the construct was evaluated using the ANTIGENpro database [29].

### 2.6 Immunofluorescence assay and western blot analysis

H1N1 epitope antigen protein expression was analyzed using immunofluorescence assay (IFA) and western blot. RAW 264.7 cells transfected with a Flag-tagged antigen construct were incubated for 48 h, fixed with 80 % acetone, permeabilized, and blocked. Primary anti-Flag mouse antibody (1:200) and FITC-conjugated secondary anti-mouse IgG1 goat antibody (1:5000) were applied, and DAPI used for nuclear staining. Results were visualized using a Leica fluorescence microscope.

### 2.7 Viral propagation, immunization, and animal challenge

The influenza A (H1N1) strains were used for viral challenges. Viral propagation was conducted in embryonated eggs (SPF) following the standard procedure. Two days after the inoculation of embryonated eggs, allantoic fluid was collected, and the viral loads were determined by TCID50 (Reed-Muench method) [30] using *in vitro* assays on MDCK cells. The viral suspensions were collected and stored at −80 °C for further utilization. Immunizations were initiated for mice when they reached six weeks old. The vaccine was given via the intramuscular route (IM). Seven mice per group were randomly allocated into four groups (Naïve, PBS, JOL3141, and JOL3143 immunized). All mice were immunized intramuscularly at 1 × 10^7^ colony-forming unit (CFU) per mouse. Booster immunization was done after 2 weeks post-primary inoculation following the same dose and route as primary immunization. At 7, 14, 21, and 28 days post-infection (dpi), four mice in each group were sacrificed for splenocyte harvest. Sera samples were also collected (n = 3) and stored at −80 °C until used. Body weight measurements were conducted in the remaining mice to assess the effects of inoculation and recorded throughout the study period. Blood samples were collected via the retro-orbital sinus following standard procedures. The harvested splenocytes were utilized for evaluating cell-mediated immune responses through splenocyte proliferation assays, flow cytometric analysis, and RNA isolation.

### 2.8 ELISA assay for determination of antibody titers

Serum samples were analyzed for total IgG subpopulations (IgG, IgG1, IgG2a, IgG2b, and IgG3) titers using ELISA. First, 96-well plates were coated with inactivated A/PR/8/34 virus in bicarbonate buffer, blocked with 5 % skim milk, and washed with PBST. Diluted serum samples were incubated for 1 h at 37 °C, followed by HRP-conjugated goat anti-mouse IgG, IgG1, IgG2a, IgG2b, and IgG3 antibodies (SouthernBiotech, AL, USA). The reaction was developed with an OPD substrate, and OD was measured at 492 nm using a Tecan microplate reader. Assays were performed in triplicate.

### 2.9 Flow cytometry and splenocyte proliferation

Two weeks post-booster, splenocytes were harvested from two mice per group and cultured in RPMI medium with 10 % FBS and 1 % penicillin-streptomycin for FACS analysis and proliferation assays. For FACS, cells were stained with PE-labeled anti-CD3e, PerCPVio700-labeled anti-CD4, and FITC-labeled anti-CD8a antibodies (Miltenyi Biotec, Bergisch Gladbach, Germany) to analyze CD3^+^CD4^+^ and CD3^+^CD8^+^ T cell subsets using the MACS Quant system. For proliferation assays, splenocytes were stimulated with inactivated virus particles for 48 h, followed by an MTT assay. The proliferation index was calculated as A570 for immunized mice divided by control mice. All experiments were performed in triplicate.

### 2.10 Quantitative real-time polymerase chain reaction

Splenocytes were isolated, seeded in 12-well plates with complete RPMI media, and stimulated with inactivated H1N1 A/PR/8/34 virus for 48 h. Total RNA was extracted using the GeneAll® Hybrid-R™ kit and reverse-transcribed into cDNA with 1 μg RNA using a Reverse Transcription Master Mix. Cytokine mRNA expression (IFN-γ, TNF-α, IL-12, IL-1β, IL-4, IL-6, IL-10, IL-8, IL-7, IL-17, and IL-23) was quantified via SYBR Green PCR with 2X Real-Time PCR Master mix (BioFACT^TM^, Daejeon, South Korea) and Applied Biosystems primers. Melting curve analysis confirmed specificity, and relative expression levels were calculated using the 2^–ΔΔCT^ method with β-actin as the internal control.

### 2.11 Hemagglutination inhibition (HI) assay

A hemagglutination inhibition (HI) assay was performed to assess the capacity of each serum sample to inhibit erythrocyte agglutination induced by virus strains. The HI assay was performed using 0.75 % chicken erythrocytes. Serum samples were heat-inactivated at 56 °C for 30 min, subjected to two-fold serial dilution, and incubated with four hemagglutination units of virus particles, as specified in the World Health Organization (WHO) manual for laboratory influenza surveillance [31]. The HI titer was determined as the reciprocal of the highest serum dilution that prevented agglutination. These assays were conducted in triplicate.

### 2.12 Microneutralization (MN) assay

The sera virus neutralization was assessed using an MN assay as previously described [32]. Heat-inactivated serum samples were subjected to two-fold serial dilution. The diluted sera were incubated with 200 TCID50 of H1N1 A/PR/8/34 for 1 h at 37 °C. Then, mixtures were subsequently added to MDCK cell monolayers in 96-well plates. The cells were incubated at 37 °C in a humidified CO_2_ incubator for 72 h and monitored daily for cytopathic effects (CPE) using a microscope. The neutralizing antibody titer was defined as the highest serum dilution that inhibited complete CPE in two out of three wells. The virus neutralization titers were also determined for the four H1N1 strains [A/H1N1/pdm09 (NCCP43400), A/Brisbane/59/2007 (NCCP42464), A/Korea/01/09 (NCCP43021), A/PR/8/34].

### 2.13 Assessment of long-term immune responses

To investigate antigen prioritization and long-term immune response, splenocytes collected in 28, 42 and 56 dpi were investigated. The collected splenocytes were stimulated with HA and NA antigens separately for 48 h. After stimulation, splenocytes were subjected to flow cytometry, MTT assay and RT-qPCR. Flow cytometry was conducted to determine activated T cell subpopulation percentage. Splenocytes were stained with antibody specific to CD3e, CD4 and CD8a T cells and subjected to MACS Quant system. The MTT assay data were presented as absorbance in wavelength 570 nm. To check immune response in 56 dpi after exposure to HA and NA antigens, we investigated the fluctuation of IFN-γ and TNF-α expression levels. Melting curve analysis confirmed specificity, and relative expression levels were calculated using the 2^–ΔΔCT^ method with β-actin as the internal control.

### 2.14 Statistical analysis

Data analysis was performed using GraphPad Prism 9.0 software (GraphPad, Boston, MA, USA). Significant differences between the vaccinated and control groups were assessed using a one-way analysis of variance (ANOVA) followed by Dunnett's multiple comparisons test. P-values less than 0.05 were considered statistically significant.

## 3 Results

### 3.1 Antigen design

**Fig. 1:**
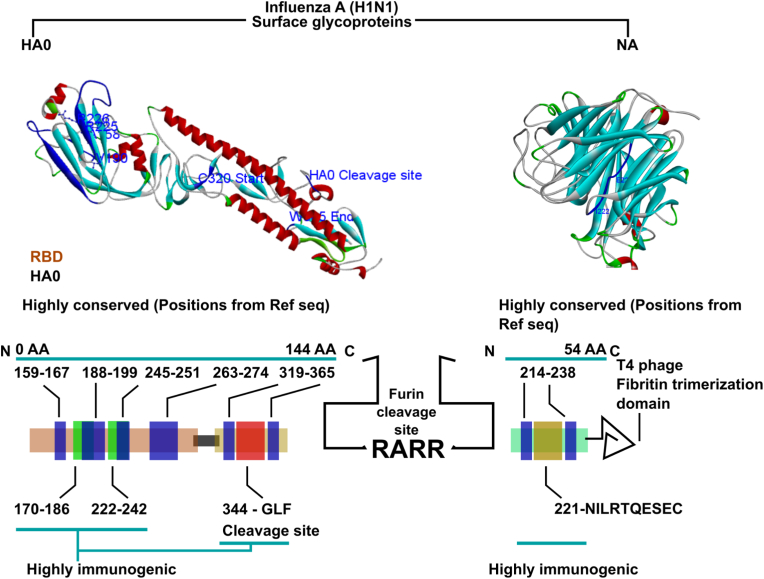
Epitope antigen protein design. HA (144 amino acids) and NA (54 amino acids) epitopes were connected with a furin cleavage site. The 144 amino acid sequence coded for the highly immunogenic HA stalk epitope, HA0 cleavage site, and five highly conserved RBD epitope regions. The 54 amino acid sequence encoded NA-derived epitope regions and a T4 phage fibritin trimerization domain. Distribution of epitope regions on the original HA and NA structures is demonstrated.

We selected HA and NA, two major proteins involved in the viral fusion and release process during the influenza life cycle, as protective antigens. HA facilitates the initial attachment to host cells with sialic acid residues during the entry stage, while NA is involved in the release of viral particles by cleaving sialic acid residues during the later stages [33]. As both HA and NA amino acid sequences are highly variable among influenza strains, we selected conserved epitopes of these proteins for *Salmonella*-mediated live attenuated vaccine development. Both HA1 and HA2 are synthesized as proteolytic cleavage precursor proteins within the endoplasmic reticulum, followed by modifications in the Golgi apparatus. Approximate sizes of the HA1 and HA2 segments are 330 and 220 amino acids, respectively. The HA1 region forms a globular head and is coded by highly variable sequences, while the stalk region found within HA2 is generally conserved. For HA antigen development, we selected regions spanning the amino acid residues 190, 225, 226, and 158, which are important residues that interact with the cell receptor. The fusion peptide region found in the stalk region also plays a pivotal role in viral fusion into the host cell membrane and is important for neutralizing antibody development. Hence, selected amino acid residues from the stalk region were included in the construct. An HA1-HA2 construct was designed containing five key immunological epitopes, namely, amino acids (AA) 159–167, 188–199, 245–251, 263–274, and 319-36 (Fig. 1). The GLF cleavage site was located between AA residues 319–365.

**Fig. 2:**
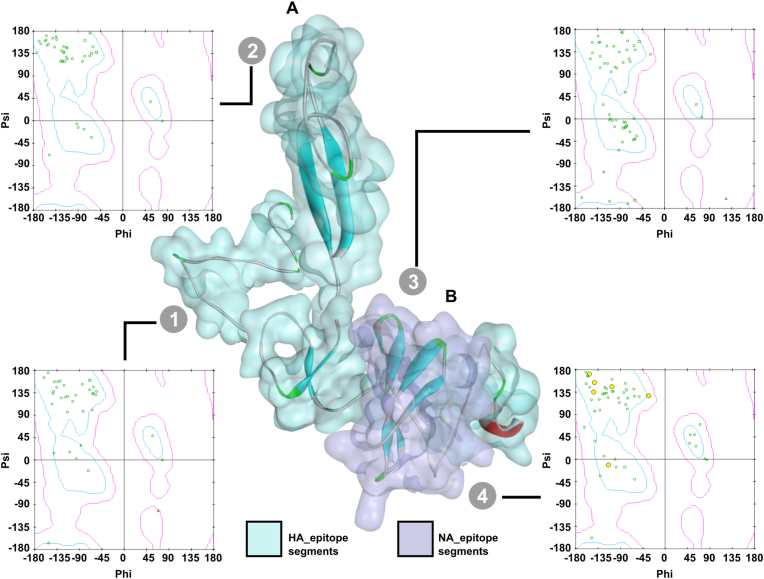
Antigen structure simulation. (A) Predicted structure of HA antigen and (B) predicted structure of NA antigen. The amino acid sequences of each protein were submitted to the SWISS model portal and structures were predicted. The full structure was developed as separate epitope sections and graphically connected into a full structure. Segment 1 consisted of epitope 1; while segment 2 consisted of epitopes 2, 3, and 4; and segment 3 consisted of epitope 5. Segment 4 depicts the NA epitope. Molecular visualization was conducted using the Discovery studio suite. Subplots are Ramachandran plots generated against each antigen.

The second protective antigen, NA, has a highly conserved epitope between 214 and 238 AA (NILRTQSESEC) that is known for its neutralization ability. Thus, this epitope region was selected and fused to the T4 fibritin domain at the C terminus for trimerization (Fig. 1). The HA construct and NA-fibrin construct were then fused with a furin cleavage sequence (RARR) to promote post-translational cleavage and facilitate antigen processing and presentation. Simulated structures of each antigen construct are provided in Fig. 2.

### 3.2 Development and verification of a vaccine construct

**Fig. 3:**
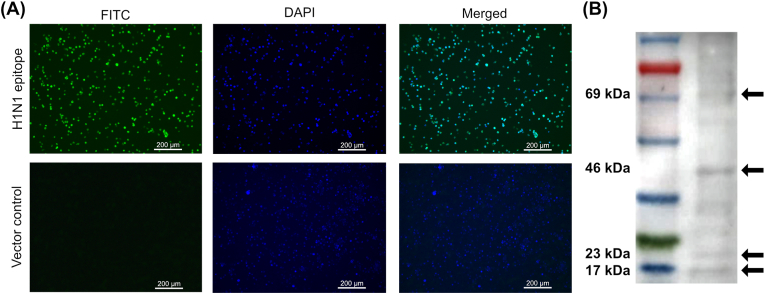
Validation of antigen protein expression. (A) Immunofluorescence assay to visualize antigen protein expression in RAW264.7 eukaryotic cells. Bright green spots (expressed antigen protein) were noted in cells transfected with the vaccine vector. (B) Eukaryotic expression was verified by western blotting using an anti-flag-tag antibody. Specific immunoreactive bands at 17.7 kDa, 23.5 kDa, 46 kDa, and 69 kDa corresponded to cleaved trimerized NA antigen, non-cleaved HA-NA, dimerized and trimerized non-cleaved HA-NA proteins, respectively. (For interpretation of the references to colour in this figure legend, the reader is referred to the Web version of this article.)

Expression of antigens by the plasmid construct was verified by IFA and western blot analysis (Fig. 3). IFA was conducted using vaccine vector-transfected RAW 264.7 cells (Fig. 3A). Antigenic epitopes tagged with flag-tag were detected in fluorescence images of transfected cells as bright green spots, corresponding to the expression of vaccine vector-induced antigens. For western blot analysis, the constructed vaccine vector (pJHL204:H1N1epi) was transfected into HeLa cells that were then incubated for 48 h to allow plasmid nuclear localization and expression. Total protein extraction and immunoblotting were conducted using flag-tag antibody. The antigen expression pattern indicated successful expression of the H1N1 epitope (Fig. 3B) by the plasmid system. The flag-tag was present at the C-terminus of the antigen, and cleaved NA antigen in trimer confirmation was visible as a 17.7 kDa band. Non-cleaved HA and NA antigen together were present at 23.5 kDa. Furthermore, dimer and trimer confirmations of non-cleaved HA-NA confirmations were represented by bands at 46 kDa and 69 kDa.

### 3.3 Salmonella-induced humoral immune responses

**Fig. 4:**
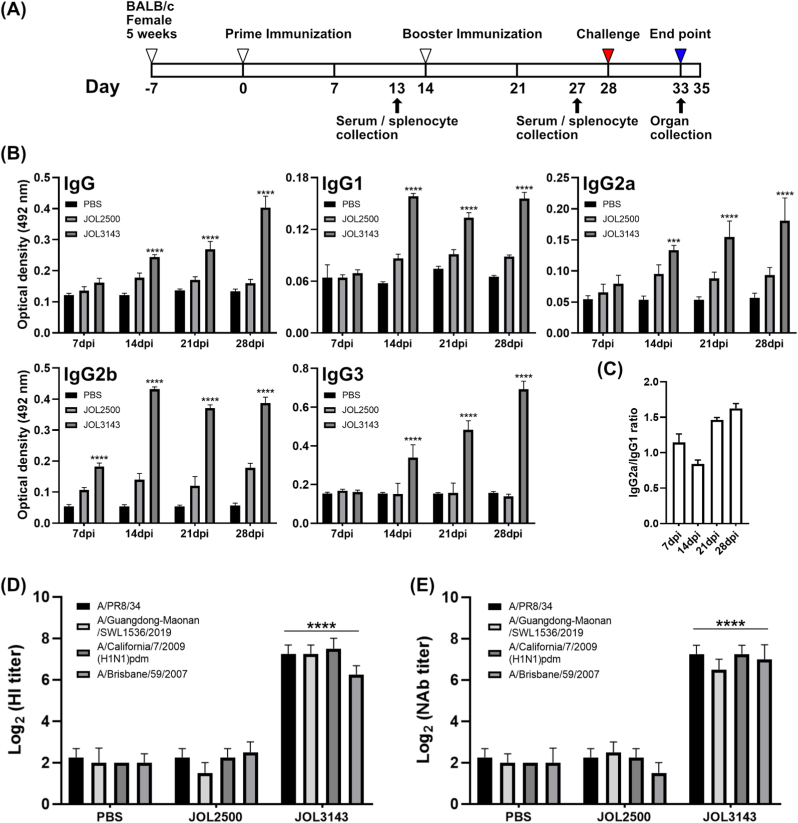
Immunization schema and humoral immune response were elicited in vaccinated mice. (A) Details of the immunization schedule. (B) Concentrations of IgG subpopulations. Sera of mice inoculated with PBS, JOL2500, and JOL3143 were collected at 7, 14, 21, and 28 dpi and measured for IgG, IgG1, IgG2a, IgG2b, and IgG3. The antibody titer was measured against inactivated A/PR/8/34 virus particles. (C) The ratio of IgG2a and IgG1 is presented to show the Th1-skewed response to immunization. (D) Serum HI titers. A serial twofold dilution of heat-inactivated serum was treated with four HA unit equivalents of the virus, and HI was assessed using chicken erythrocytes. (E) Neutralization assay of serum samples from immunized mice. Sera were collected at 28 dpi, and the NAb titer was measured in MDCK cells as inhibition of CPE at maximum dilution. Level of significance was set to P < 0.05. Asterisks indicate significant differences compared to the respective PBS control.

Serum samples were collected to assess humoral immune responses (Fig. 4A). Two weeks after primary and booster immunizations, immunized mice exhibited significant increases in serum IgG levels (Fig. 4B). Notably, JOL3143-immunized mice demonstrated antigen-specific humoral responses 2 weeks after primary inoculation compared to the JOL2500 (vector control) group. IgG1 and IgG2a subtypes followed a similar trend, peaking at 2 weeks after primary immunization with a consistent IgG1/IgG2a ratio within the 0.5–2.0 range, indicating balanced Th1 and Th2 immune responses (Fig. 4C). ELISA analysis of sera collected at 14, 21, and 28 dpi showed elevated IgG subtypes in all except the PBS group. JOL3143 immunization significantly increased IgG1 level, indicating a strong Th2-mediated response in early immune induction (14 dpi), followed by an increase in IgG2a level, suggesting enhanced Th1-mediated immunity. Activation of both Th1 and Th2 arms of protective immunity by immunization could be particularly important. A balance in immune responses is important to prevent detrimental outcomes. A particular skew toward Th1 type immunity, which has a rigorous antiviral effect, was evident at 21 and 28 dpi. Elevated IgG3 level was observed in the JOL3143-immunized group (Fig. 4B). Because the IgG3 subtype recognizes a broad antigen spectrum more efficiently than other IgG subtypes, it is important for recognition of antigenically drifted influenza viruses [34].

Sera from JOL3143-immunized mice demonstrated cytopathic effect (CPE) inhibition, with an average neutralizing antibody (NAb) titer of log2(7), which was significantly higher than that of the naïve and vector control groups (Fig. 4D). Hemagglutination inhibition (HI) assays revealed higher titers in JOL3143-immunized mice than controls, confirming the generation of potent antibodies (Fig. 4E). Humoral immunity induced by JOL3143 effectively neutralized multiple virus strains.

### 3.4 Antigen-specific cellular immunity

**Fig. 5:**
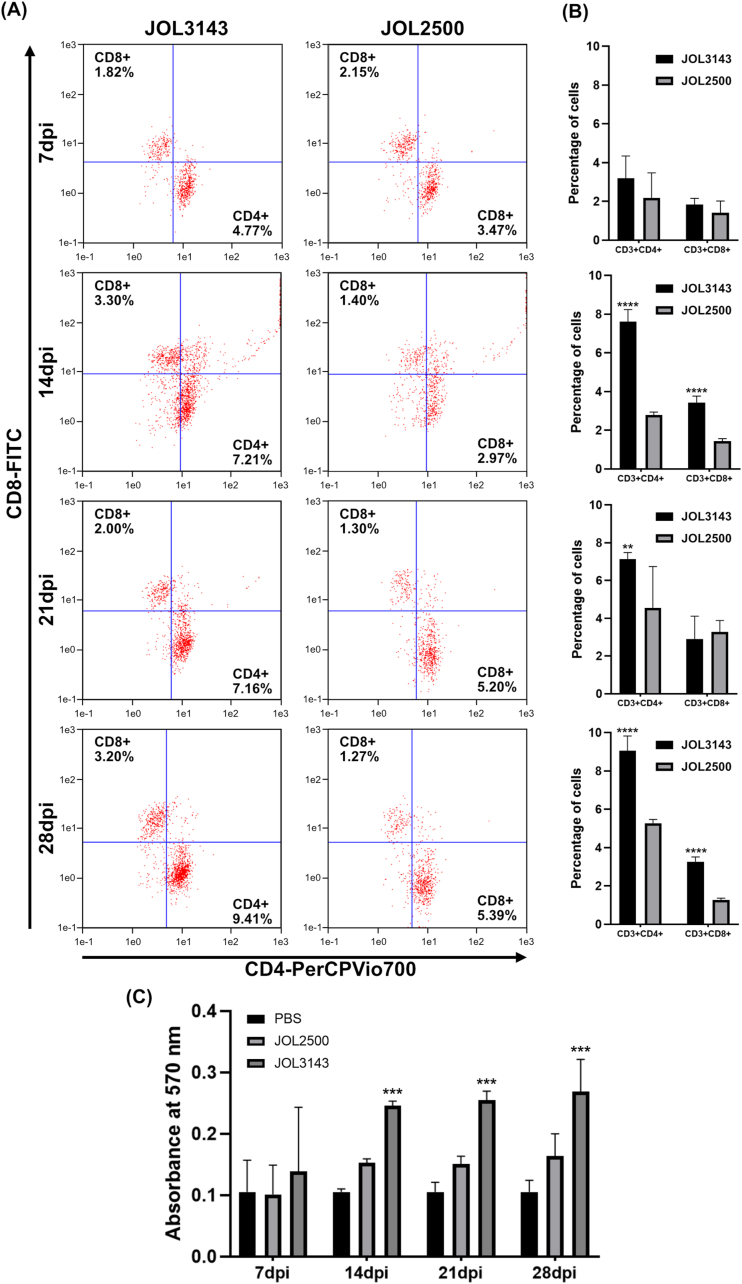
T cell-mediated immune response in splenocytes. (A, B) Data represent changes in the percentages of CD3^+^CD4^+^ and CD3^+^CD8^+^ T cells gated in CD3^+^ in response to stimulating splenocytes with inactivated virus particles. (C) Proliferation index of stimulated splenocytes. Level of significance was P < 0.05. Asterisks indicate significant differences compared to the respective JOL2500 group.

Evaluation of the T-cell-mediated immune response was performed after initial and booster immunization (Fig. 5). Antigen-specific CD3^+^CD4^+^ and CD3^+^CD8^+^ T-cell responses were evident in JOL3143-immunized mice (Fig. 5A and B). Initial immunization resulted in immune responses at 14 dpi, indicating delayed antigen recognition. The booster-immunized group demonstrated a faster response within 7 days after the second exposure and a stronger response at 28 dpi. A significant increase in CD4^+^ and CD8^+^ populations was observed, with a significant skew toward CD3^+^CD4^+^ responses. These observations are consistent with a prominent Th2 immune response, which is observed in humoral immune responses. Furthermore, splenocyte proliferation demarcated significant engagement of cell-mediated immune responses in JOL3143-immunized mice compared to the vector control (Fig. 5C). A high absorbance in MTT assay results indicated robust engagement of the immune system upon re-encounter of the target antigens or viral particles.

### 3.5 Cytokine responses

**Fig. 6:**
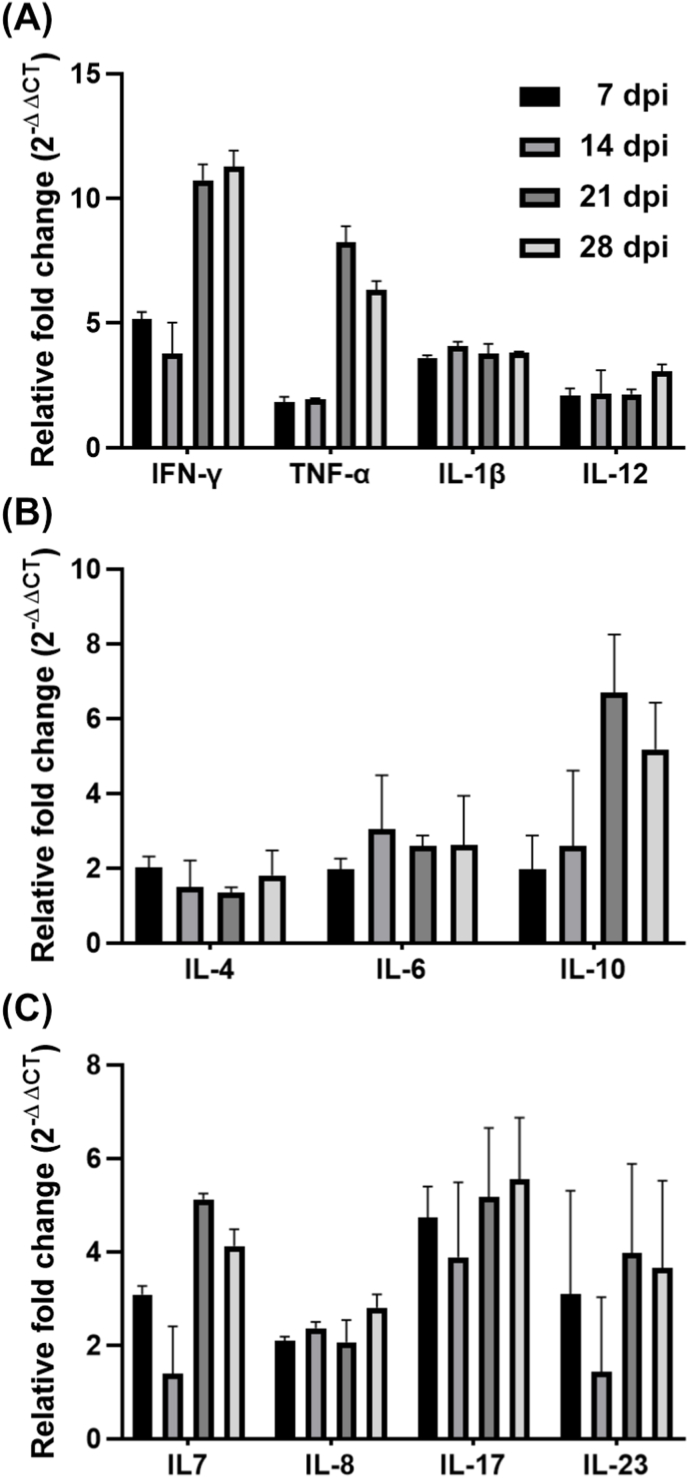
Cytokine expression levels. Relative fold changes in the expression of cytokines were determined by qRT-PCR 48 h after stimulating splenocytes with inactivated virus particles. Changes in cytokine levels were measured at the mRNA transcript level using the 2^–ΔΔCT^ method, with β-actin as the internal control. (A) Th1-related cytokine expression levels. (B) Th2-related cytokine expression levels. (C) Expression levels of cytokines were non-specifically associated with Th1 and Th2 responses.

Immunomodulatory cytokine transcription levels were assessed via qRT-PCR (Fig. 6). Levels of Th1-related cytokines (IFN-γ, TNF-α, IL-12, and IL-1β) were significantly elevated, indicating a strong cell-mediated immune response after vaccination, especially with the booster dose (Fig. 6A). High IFN-γ expression indicated activation of cellular immunity demarcated by CD8^+^ T cell responses. TNF-α upregulation results in activation of macrophages and neutrophils, critical for cell-mediated immunity, while IL-12 promotes naïve T cell differentiation into Th1 cells. Th2-related cytokines (IL-4, IL-6, and IL-10) were also upregulated overall after immunization (Fig. 6B). Marginal increases in IL-4 and IL-6 expression were evident. IL-6 assists in antibody production and B cell activation, while IL-10 promotes humoral immunity by aiding B cell differentiation and suppressing excessive inflammation. Non-specific cytokines (IL-8, IL-7, IL-17, and IL-23) also showed increased expression (Fig. 6C). Elevated IL-8 levels indicated acute inflammation, neutrophil recruitment, and innate immunity, which we considered to be induced by the bacterial vaccine vehicle. Higher IL-7 levels suggested enhanced T-cell survival and long-term immune memory induced by booster immunization. Th17-associated cytokines (IL-17) were also upregulated, indicating Th17 cell activation. Elevated IL-23 suggested strong differentiation and maintenance of Th17 cells, which are crucial for mucosal immunity against respiratory pathogens.

### 3.6 Challenge-protection

**Fig. 7:**
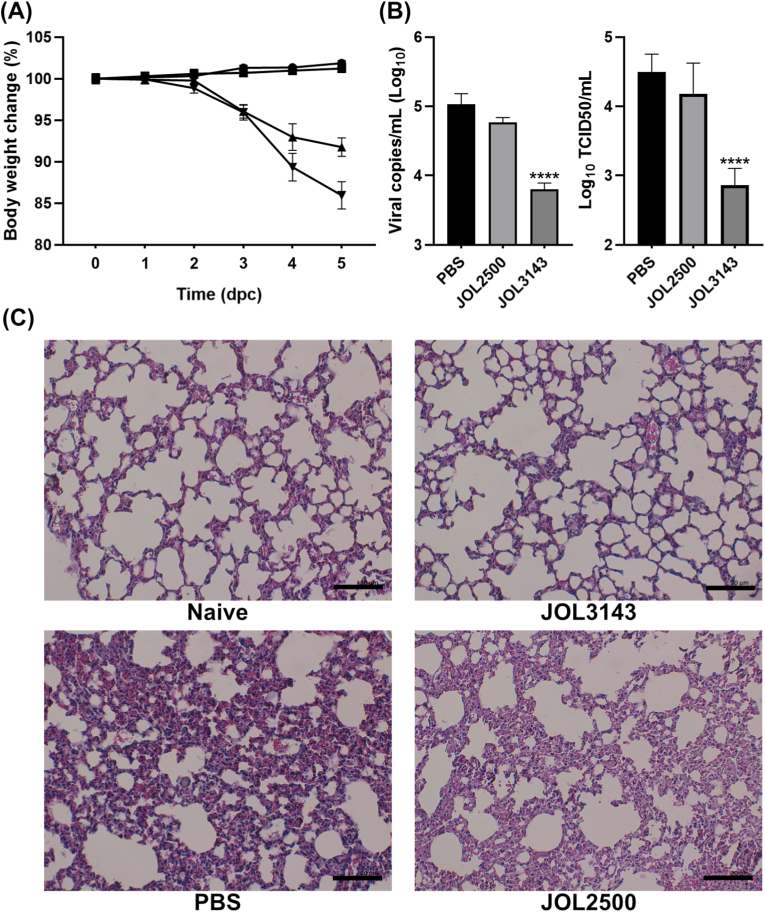
*In vivo* evaluation of the protective effects of the vaccine against influenza A/PR/8/34 virus. (A) Body weight changes monitored up to 5 dpc. (B) Viral copy number measured 5 dpc. (C) Viral load determination using TCID50 methods. (D) Histopathological analysis of the protective efficacy of JOL3143. Lung samples collected at 5 dpc were H&E stained and analyzed. Inflammation and alveolar collapse were denoted in PBS and JOL2500-immunized groups. Level of significance was P < 0.05. Asterisks indicate significant differences compared to the PBS control.

**Fig. 8:**
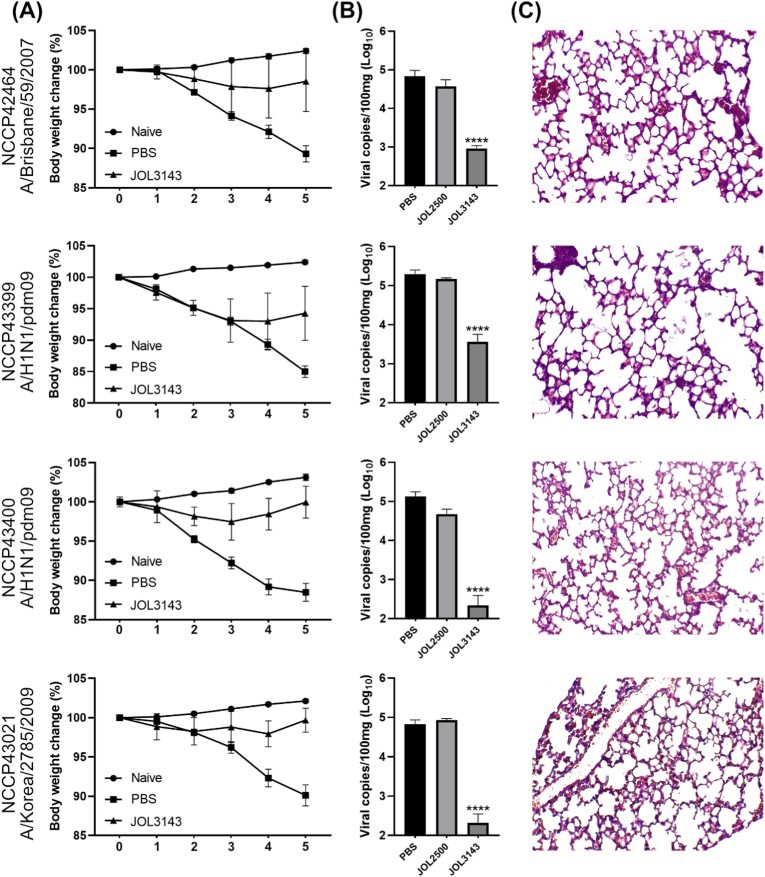
*In vivo* evaluation of protection against influenza A/H1N1/pdm09 (NCCP43399 and NCCP43400), A/Brisbane/59/2007 (NCCP42464), A/Korea/01/09 (NCCP43021) virus strains. (A) Body weight changes monitored up to 5 dpc. (B) Viral copy number measured at 5 dpc. (C) Histopathological analysis of the protective efficacy of JOL3143. Lung samples collected at 5 dpc were H&E-stained and analyzed. Level of significance was P < 0.05. Asterisks indicate significant differences versus the PBS controls.

A challenge assay was conducted to evaluate the *in vivo* protective efficacy of JOL3143 (Fig. 7, Fig. 8). Fourteen days post-booster immunization, mice were intranasally challenged with influenza viruses and sacrificed at 5 dpc. Body weight was monitored as a measure of systemic infection. In response to the A/PR/8/34 challenge, the PBS-immunized group exhibited marked weight loss, whereas mice immunized with JOL3143 maintained a stable body weight (Fig. 7A). Correspondingly, viral load and copy numbers were significantly reduced in the JOL3143 group compared to PBS controls (Fig. 7B and C). Histopathological analysis demonstrated that JOL3143 mitigated the characteristic lung pathology associated with H1N1 infection (Fig. 7C). Severe pulmonary inflammation, including diffuse infiltration of inflammatory cells, alveolitis, and alveolar collapse, was observed in PBS- and JOL2500-immunized mice. These features were substantially diminished in JOL3143-immunized mice, which displayed preserved alveolar architecture and reduced inflammatory cell infiltration.

To assess cross-protective efficacy, mice were also challenged with four additional H1N1 strains (NCCP43399, NCCP43400, NCCP42464, and NCCP43021) (Fig. 8). While mild body weight loss occurred, recovery was evident by 4 dpc (Fig. 8A). Viral copy numbers were significantly lower in the JOL3143 group than the vector control and PBS groups (Fig. 8B). Histological evaluation confirmed broad protection against lung pathology induced by diverse viral strains (Fig. 8C). These findings demonstrate that JOL3143 confers strong *in vivo* protection against systemic and respiratory influenza infection with multiple H1N1 strains.

### 3.7 Assessment of long-term immune responses

**Fig. 9:**
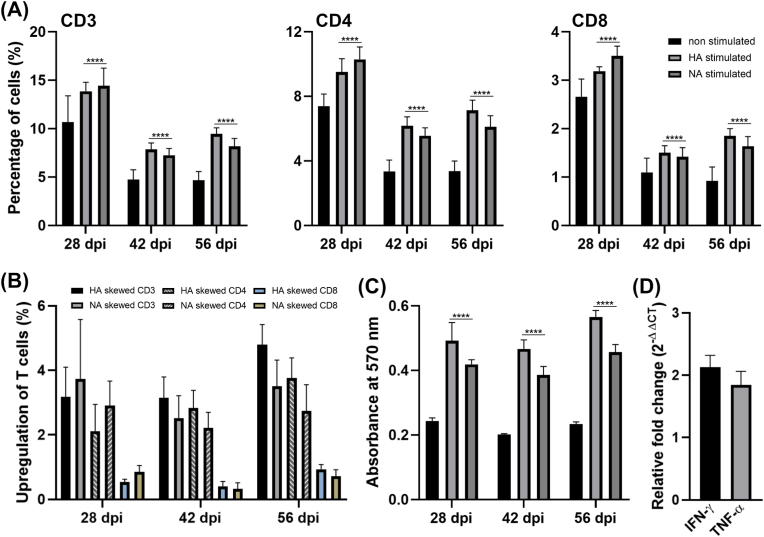
Long-term immune response stimulated with HA and NA antigen proteins. (A) The percentages of CD3^+^, CD4^+^ and CD8^+^ T cells in response to stimulating splenocytes with HA and NA proteins. (B) Elevation of T cell subpopulation after HA and NA stimulation compared to non-stimulated splenocytes. (C) MTT assay of splenocytes after HA and NA stimulation. (D) The expression levels of IFN-γ and TNF-α in splenocytes, collected in 56 dpi, after HA and NA stimulation. Level of significance was P < 0.05. Asterisks indicate significant differences versus the non-stimulated group.

We performed additional assays to investigate long-term immunity and antigen priority (Fig. 9). The splenocytes collected in 28, 42 and 56 dpi showed significant elevation of T cell subpopulations. Flow cytometry analysis of CD3^+^, CD4^+^, and CD8^+^ T cell responses showed a peak at two weeks post-booster (28 days post-immunization) and gradually declined by 28 days after the booster (42 days post-immunization) while maintaining a basal level of immune response, still significant compared to the non-stimulated cells. These responses persisted up to 56 days post-booster, indicating memory responses were maintained until at least 42 days after boosting. Harvested splenocytes responded significantly to both HA and NA antigens, suggesting that each antigen was recognizable by the immune system sensitized through *Salmonella* immunization. The MTT assay results also demonstrated similar patterns (Fig. 9C). The IFN-γ and TNF-α expression levels also indicated the robust immune response in stimulated splenocytes collected in 56 dpi (Fig. 9D).

## 4 Discussion

We developed an effective vaccine model against influenza A (H1N1) by combining an engineered *Salmonella* strain with attenuated virulence, an efficient RdRp-driven eukaryotic plasmid system, and a specially designed HA and NA epitope construct. The vaccine modality targets the H1N1 serotype, as this serotype is responsible for more than 70 % of all reported human influenza cases [35,36]. Development of effective immunization efforts is of great importance in the modern era where pandemic risk is high due to high population density and environmental changes. Currently available commercial vaccines are either inactivated viruses (Fluarix by GlaxoSmithKline) or subunits (Flublok by Sanofi Pasteur); however, no existing vaccine model utilizes *Salmonella* or any other bacterium as a carrier agent. To address issues of existing vaccine models related to depth and breadth of protection, we employed two key protective influenza immunogens, HA and NA, which we fused to form a combined structure. Both HA and NA are antigens [37] involved in viral attachment and release during the viral life cycle, and the World Health Organization (WHO) recommends incorporating these into vaccines. To prevent invasive viral particles, we focused on the HA antigen, especially the RBS. One significant issue associated with RBS is its significant sequence variability due to naturally occurring mutations, giving rise to novel strains. To capture the conserved epitopes surrounding the RBS, we selected five epitopes, including components from RBS and the GLFd cleavage site at the C-terminus of the HA antigen. To enhance the stability of the HA structure, components of the globular head (HA1) and the stalk region (HA2) were utilized. To promote antigen processing, addition of a furin cleavage site is advantageous, as cellular proteases can fraction large proteins into smaller segments that can be easily processed and presented via major histocompatibility complexes I and II for antigen display on cellular membrane structures. This approach can also promote a broader array of small antigen segments that are available for the immune process (Fig. 1) [38]. NA has a central core epitope known to elicit protective immune responses. The smaller NA fragment was fused with the T4 fibritin domain to promote trimerization, which is favorable for antigen presentation. Naturally, NA remained a tetramer; however, we did not assume that simple epitopes of this protein would successfully adopt a tetramer conformation. We hypothesized that presentation of NA as a trimer would lead to an improved immune response than it remains as a single epitope construct (Fig. 1). Several concerns could arise for utilizing the T4 fibritin domain, as it may give rise to T4 domain-specific adverse immune responses. However, in the current investigation, we confirmed that the current vaccine design delivered by the *Salmonella* system did not cause any significant health-related concerns in immunized mice.

Strategically, delivering influenza HA and NA epitopes (Fig. 2) via the *Salmonella* system directly targeted the host's antigen presentation cells, conferring several advantages in terms of the immune process. *Salmonella* protects antigens from hostile environments encountered during inoculation, prolongs the immune elicitation time when *Salmonella* is present in the inter- and intracellular environments, and is efficiently circulated within the lymphatic system as the *Salmonella* system is highly proficient at targeting immunological niches such as mesenteric lymph nodes (MLNs) and the gut-associated lymphatic system (GALT) [39]. In the present study, we confirmed that our novel *Salmonella* system could deliver antigens into epithelial (HeLa) cells and phagocytic (RAW 264.7) cells using *in vitro* immunofluorescence assays (Fig. 3A) and western blot analysis (Fig. 3B). Our results demonstrate that the phage T4 domain could effectively maintain a trimer structure, while a proportion of antigen was broken into smaller fragments due to the activity of furin proteases.

To confirm that the delivered antigens were inducing the intended protective immune responses, we conducted an immunization and challenge study. We employed BALB/c mice inoculated with the therapeutic *Salmonella* strain code-named JOL3143 and compared the PBS and vector-only groups. Successful antigen-specific IgG humoral responses were generated, demonstrating a boost during the prime-boost immunization schedule. The inoculation route was IM, as this route promotes rapid and uniform absorption, offers high bioavailability, and has the capacity to hold large antigen volumes with prolonged action [40]. As an intracellular pathogen, *Salmonella* has a reputation for eliciting strong cell-mediated immune responses involving multiple cell types in the immune system. This key adaptive response eliminates these pathogens from the host environment. However, immune profile data suggested that the current *Salmonella* vaccine confers a balance of cellular-humoral immune responses marked by IgG1 and IgG2a ratios (Fig. 5A, B, and 5C) and CD4^+^/CD8^+^ T cell responses (Fig. 5A and B) [41]. We also evaluated fluctuations in IgG3 antibody responses and found a remarkable increase on days 14, 21, and 28 post-immunization (Fig. 5B). Previous studies described the various roles of IgG subtypes in antigen recognition and viral infection stages [34,[42], [43], [44]]. IgG3 is closely associated with the primary response against viral infections and recognition of drifted virus antigens. The significant IgG3 elevation noted in our study likely contributed to cross-protection against challenge with several H1N1 influenza strains (Fig. 8). These antibody responses could confer essential broad protection; we found that they could neutralize several other H1N1 strains, including A/California/7/2009 and A/Brisbane/59/2007 (Fig. 4D and E). These humoral immune response effects could be attributed to the contributions of HA and NA antigens, which are proficient humoral immune inducers, and could be specific to the current vaccine scenario. Balanced immune responses are important for safety aspects of the current vaccine model, as these responses can minimize detrimental inflammatory responses elicited by bacterial components such as lipopolysaccharides (LPS) [45]. Moderate inflammation elicited by PAMPs could be beneficial for safe elimination of viral infection due to controlled activation of type I interferon markers; for example, by the cGAS-STING pathway, and elicitation of IFN-α and IFN-β [46]. These responses could boost strong antiviral activity to suppress influenza infections.

The cytokine profiles shown in Fig. 6 demonstrate a well-coordinated immune response induced by the JOL3143 vaccine. Th1 cytokines (Fig. 6A), including IFN-γ, TNF-α, IL-1β, and IL-12, were strongly upregulated—particularly at 14 and 21 dpi—indicating robust cell-mediated immunity involving CD8^+^ T cell activation and macrophage recruitment [47,48]. These findings are consistent with activation of innate antiviral signaling pathways, amplifying antiviral activity in early infection stages. Moderate increases in Th2 cytokines (Fig. 6B) such as IL-4, IL-6, and IL-10 suggest supportive roles in humoral immunity and inflammation regulation, with IL-10 peaking at 21 dpi, likely mitigating vaccine vector-associated inflammation. Additionally, elevated levels of IL-7, IL-8, IL-17, and IL-23 (Fig. 6C) highlight enhanced T cell memory, acute innate responses, and sustained Th17 activity, all of which are essential for mucosal protection against respiratory pathogens. These cytokines, particularly IL-23, support sustained Th17 activity, critical for bridging innate and adaptive immunity at mucosal surfaces [49]. Together, these results suggest that JOL3143 induces a finely tuned immune response that balances antiviral efficacy with inflammation control. The coordinated activation of Th1, Th2, and Th17 pathways, along with regulatory cytokines, points to a promising vaccine platform capable of providing robust and durable protection against influenza while minimizing adverse inflammatory effects.

The *Salmonella-*mediated HA-NA epitope vaccine model significantly protected immunized mice against lethal challenge with the H1N1 strain. Immunized mice did not show observable behavioral changes or continuous weight reduction compared to the control mice, while exhibiting significantly reduced viral copy numbers in their lungs compared to the PBS controls (Fig. 7, Fig. 8B). Despite the mismatch between the immunization route (IM) and the challenge route (nasal), the *Salmonella*-based vaccine was able to elicit systemic immune responses strong enough to suppress influenza activity and protect mice with minimal pathological effects. The extent of protection was evident in H&E-stained post-challenge lung tissues, where the immunized lungs appeared near normal in terms of tissue architecture and inflammation (Fig. 7, Fig. 8C). Furthermore, serum collected from immunized mice exhibited the ability to neutralize closely related influenza H1N1 strains. This cross-protection ability is an advantage of our *Salmonella-*mediated influenza vaccine model to making it more effective against a wider range of H1N1 viruses. Furthermore, we have confirmed that the mice immunization with the current therapeutic *Salmonella* could mount a long-lasting immune response, as we investigated the efficient cellular responses even after 42 days (56 dpi) from the last inoculation (Fig. 9). This is particularly important for mitigating seasonal influenza, especially in resource-limited developing regions of the world.

## 5 Conclusions

The results of this study underscore the potential of epitope-based vaccine designs integrated into a *Salmonella* system to elicit a robust, multifaceted, and synergistic immune response, even in the absence of large protein antigens. *Salmonella's* inherent immune-stimulating properties amplified the protective effects of HA and NA epitopes, effectively engaging both innate and adaptive immunity for comprehensive protection. Moreover, this system is not only highly effective but is also scalable, enabling rapid vaccine deployment—a critical advantage in response to influenza outbreaks.

## CRediT authorship contribution statement

**Jun Kwon:** Writing – review & editing, Writing – original draft, Methodology, Investigation, Data curation, Conceptualization. **Amal Senevirathne:** Writing – review & editing, Writing – original draft, Methodology, Investigation, Data curation, Conceptualization. **John Hwa Lee:** Writing – review & editing, Supervision, Funding acquisition.

## Funding

This work was supported by the 10.13039/501100003725National Research Foundation of Korea (NRF) grant funded by the Korea government (10.13039/501100014188MSIT) (No. RS-2023-00272216).

## Declaration of competing interest

The authors declare that they have no known competing financial interests or personal relationships that could have appeared to influence the work reported in this paper.

## Data Availability

Data will be made available on request.
