# Supplementary material for: Harnessing Salmonella as a potent vaccine delivery platform: Targeted HA/NA epitope presentation via alphavirus RdRp-driven expression to boost immune efficacy
Source: Mater Today Bio. 2025 Aug 21;34:102224. doi: 10.1016/j.mtbio.2025.102224 (PMC12446767; doi:10.1016/j.mtbio.2025.102224)
Supplement: Multimedia component 1 [file mmc1.docx]

**<Research Article>**

Harnessing *Salmonella* as a Potent Vaccine Delivery Platform: Targeted HA/NA Epitope Presentation via Alphavirus RdRp-Driven Expression to Boost Immune Efficacy

Jun Kwon^1^, Amal Senevirathne^1^, John Hwa Lee*

Laboratory of Veterinary Public Health, College of Veterinary Medicine, Jeonbuk National University, 79 Gobong-ro, Iksan, 54596, Jeollabuk-do, Republic of Korea

^1^These authors equally contributed to this work.

*Correspondence

e-mail: johnhlee@jbnu.ac.kr, Tel: +82-63-850-0940

**Table S1. Bacteria, plasmid, and primers used in this study**

| Bacteria/plasmid/primers | | Genotypic characteristics | References |
| --- | --- | --- | --- |
| *Salmonella* *typhimurium* | | | |
| JOL2500 | | Δ*lon*, Δ*cpxR*, Δ*sifA*, Δ*asd* | Lab stock |
| JOL3143 | | JOL2500 carrying pJHL204:H1N1 epitope | This study |
| *Escherichia coli* | | | |
| *Escherichia coli* 232 | | F- λ- φ80Δ(lacZYA-argF) endA1 recA1 hadR17 deoR thi-1 glnV44 gyrA96 relA1 ΔasdA4 | Lab stock |
| JOL3141 | | *Escherichia coli* 232 carrying pJHL204:H1N1 epitope | This study |
| Plasmid | | | |
| pJHL204 | | asd+, CMV promoter, SV40 promoter, pBR322 ori | Lab stock |
| Primers | |  |  |
| *TNF-α* | Sense | CATCTTCTCAAAATTCGAGTGACAA |  |
|  | Antisense | TGGGAGTAGACAAGGTACAACCC |  |
| *IFN-γ* | Sense | TCAAGTGGCATAGATGTGGAAGAA |  |
|  | Antisense | TGGCTCTGCAGGATTTTCATG |  |
| *IL-10* | Sense | CCTGAGCAGGATGGAGAATTACA |  |
|  | Antisense | TCCAGAACATGCCGCAGAG |  |
| *IL-6* | Sense | GGAGGCTTAATTACACATGTT |  |
|  | Antisense | TGATTTCAAGATGAATTGGAT |  |
| *IL-17* | Sense | ACCGCAATGAAGACCCTGAT |  |
|  | Antisense | TCCCTCCGCATTGACACA |  |
| *IL-1β* | Sense | TTCACCATGGAATCCGTGTC |  |
|  | Antisense | GTCTTGGCCGAGGACTAAGG |  |
| *β-actin* | Sense | AGAGGGAAATCGTGCGTGAC |  |
|  | Antisense | CAATAGTGATGACCTGGCCG |  |
| H1N1 epitope | Sense | TGAGGATCGTTTCGC GGGCCC GCCGCCACCATGTATAGCG |  |
|  | Antisense | AAGGCGCGCCTGATCA GGGCCC TTACAGAAAGGTGCTCAGC |  |
| H1N1 epitope Flag tag | Antisense | AAGGCGCGCCTGATCA GGGCCC TTACTTGTCGTCATCGTCTTTGTAGTCCAGAAAGGTGCTCAGC |  |


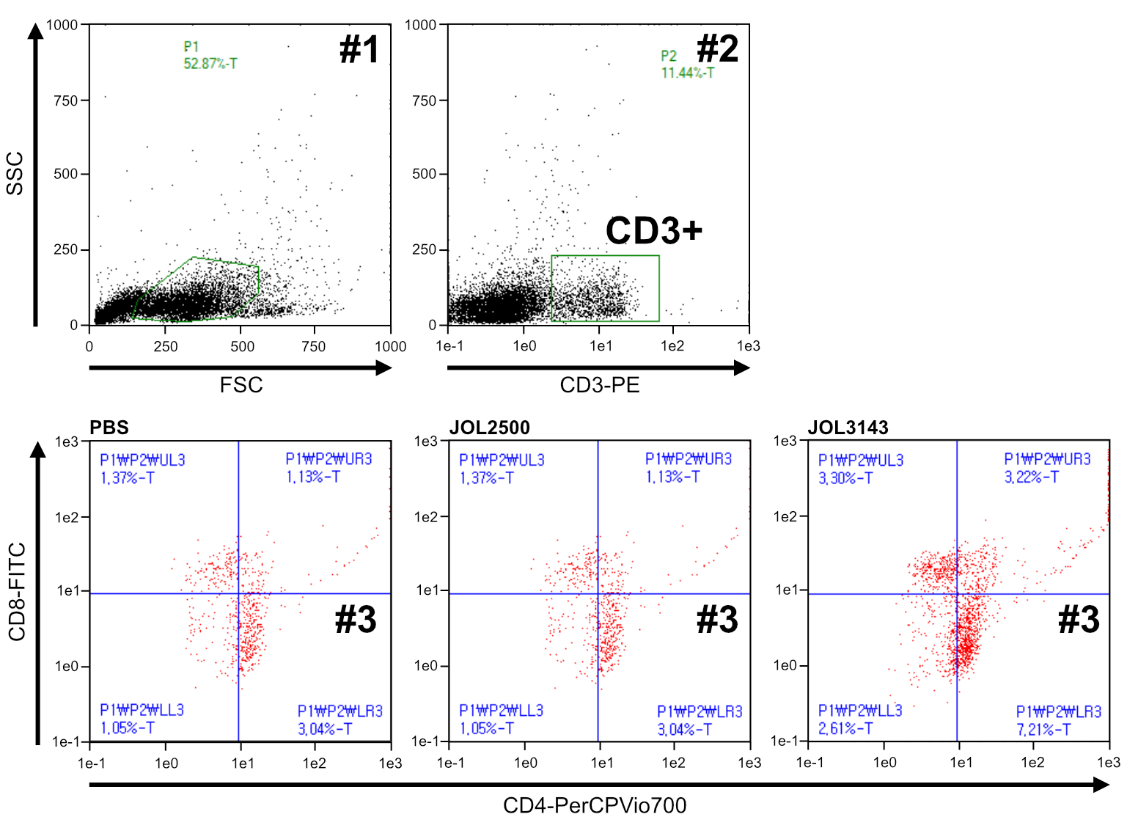


**Figure S1. Gating strategy for T cell subset population.** Splenocytes stimulated with inactivated viral particles were stained for T lymphocyte subsets with PE-labeled anti-CD3e, PerCPVio700-labeled anti-CD4, and FITC-labeled anti-CD8a anti-mouse antibodies. Cells were counted with flow cytometry, and CD3^+^ T cells were gated on total lymphocytes. From the gated CD3^+^ T cells, CD4^+^ and CD8^+^ T cells were separated based on the conjugated fluorescence colors. The panel illustrates a strategic gating approach in order, from #1 to #3, to separate CD4^+^ and CD8^+^ T cells from lymphocytes.
